# Analysis of Serum Proteome after Treatment of Osteoporosis with Anabolic or Antiresorptive Drugs

**DOI:** 10.3390/metabo12050399

**Published:** 2022-04-28

**Authors:** Alvaro del Real, Sergio Ciordia, Carolina Sañudo, Carmen Garcia-Ibarbia, Adriel Roa-Bautista, Javier G. Ocejo-Viñals, Fernando Corrales, Jose A. Riancho

**Affiliations:** 1 Departamento de Medicina y Psiquiatría, Universidad de Cantabria, IDIVAL, 39008 Santander, Spain; delreala@unican.es (A.d.R.); carolinasanudo@gmail.com (C.S.); carmen.garciai@scsalud.es (C.G.-I.); 2 Laboratorio de Proteómica Funcional, Centro Nacional de Biotecnología-CSIC, Proteored-ISCIII, 28049 Madrid, Spain; proteomics.masas@cnb.csic.es (S.C.); fcorrales@cnb.csic.es (F.C.); 3 Servicio de Medicina Interna, Hospital U. Marqués de Valdecilla, 39008 Santander, Spain; 4 Servicio de Inmunología, Hospital U. Marqués de Valdecilla, IDIVAL, 39008 Santander, Spain; adrielantonio.roa@scsalud.es (A.R.-B.); javiergonzalo.ocejo@scsalud.es (J.G.O.-V.)

**Keywords:** proteome, osteoporosis, bone, antiresorptive drugs, teriparatide

## Abstract

The aim of the study was to explore new markers in serum proteome associated with the response to antiosteoporosis drugs, namely teriparatide and denosumab. We obtained serum samples from 14 patients with osteoporosis, both at baseline and after 6 months of treatment with teriparatide (*n* = 10) or denosumab (*n* = 4). Samples were analyzed by nanoliquid chromatography coupled to high-resolution mass spectrometry on a QTOF 5600 (SCIEX) apparatus. The spectrometry data were analyzed with Mascot against the UniProtKB base and then several quality-control filters were applied for the identification of peptides (false discovery rate, FDR q < 0.02) and their quantification (FDR q < 0.05). In the group treated with teriparatide, 28 proteins were identified with significant differences before and after treatment. A pathway analysis by using the Reactome database revealed significant enrichment in the Insulin Like Growth Factor 1 (IGF-I) (FDR q 4 × 10^−2^) and innate immune system (FDR q 2 × 10^−3^) pathways. Among patients treated with denosumab, we observed significant differences in the levels of 10 proteins, which were also enriched in the pathways related to the innate immune system (FDR q 3 × 10^−2^). These results suggest that the innate immune system may be involved in the response to antiosteoporosis drugs.

## 1. Introduction

Osteoporosis is a skeletal disorder characterized by low bone mass and increased susceptibility to fracture. The disease is caused by an imbalance in bone remodeling, with an exaggerated bone resorption and/or an insufficient bone formation, that result in reduced bone mineral density (BMD); microarchitectural damage of bone tissue and decreased skeletal strength. Thus, drugs used to prevent or treat osteoporosis are classified according to their mechanism of action, as either antiresorptive or anabolic. Among antiresorptive drugs, bisphosphonates and denosumab are the most widely used in clinical practice; whereas molecules structurally related to parathormone (PTH), such as teriparatide and abaloparatide, are anabolic (i.e., bone-forming) agents frequently used to treat osteoporosis [1,2].

Osteoclasts and osteoblasts are the main cells responsible for bone resorption and bone formation, respectively. Denosumab is a monoclonal antibody that blocks Receptor Activator for Nuclear Factor κ B Ligand (RANKL), an important driver of osteoclast differentiation. Anabolic drugs stimulate osteoblast differentiation, but the mechanism of action is incompletely known.

Changes in bone remodeling are mirrored by changes in the serum levels of several biochemical bone turnover markers (BTM). Some markers [such as, Tartrate-resistant acid phosphatase (TRAP) or C-terminal telopeptide of type 1 collagen (CTX)] reflect bone resorption, whereas others [such as alkaline phosphatase or Procollagen type I N-terminal propeptide (P1NP)] reflect bone formation. Those markers are either products of bone cells (alkaline phosphatase, TRAP, P1NP) or result from the degradation of bone matrix during bone resorption (CTX). In keeping with this, therapy with anabolic or antiresorptive drugs induce changes in the serum levels of BTM, as predicted by their mechanism of action [3]. However, very few studies have comprehensively explored the association of serum proteome patterns with osteoporosis or the response to antiosteoporotic drugs [4,5]. Therefore, we planned this proof-of-concept exploratory study with the aim of analyzing the impact of drug therapy on serum peptides using a hypothesis-free approach.

## 2. Results

### 2.1. Teriparatide

**Figure 1:**
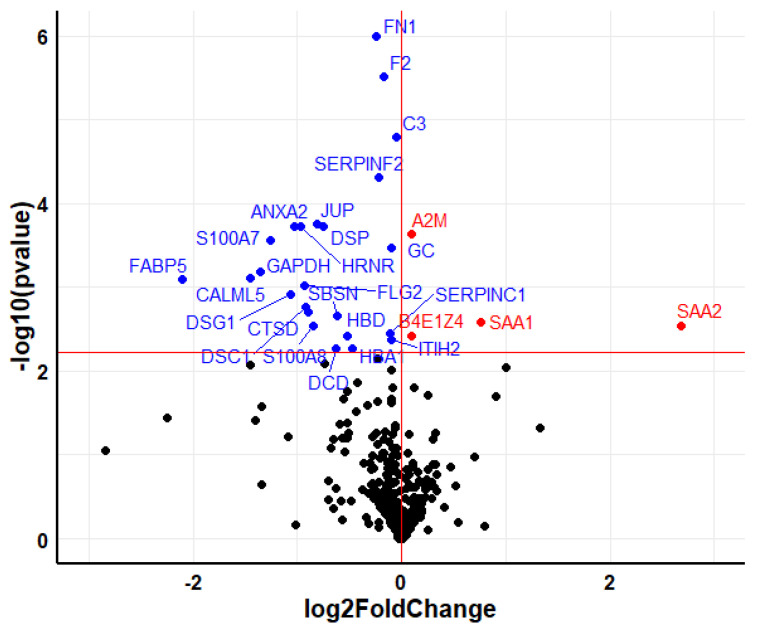
Volcano plot showing the differentially expressed proteins in serum after 6 months of treatment with teriparatide. The upregulated proteins are colored in red, whereas the downregulated proteins are colored in blue. Horizontal red line marks the q-value threshold (0.05). Black circles are those proteins under the q-value threshold (not significant).

Among the 10 paired serum samples, before and after treatment with teriparatide, a total of 621 proteins were identified. After excluding overwhelming proteins, such as keratins and apolipoproteins, 28 proteins showed statistically significant differences when baseline and post-therapy levels were compared (Figure 1).

The genes encoding the 28 differentially expressed proteins, ordered by q-value, are shown in Table 1.

**Figure 2:**
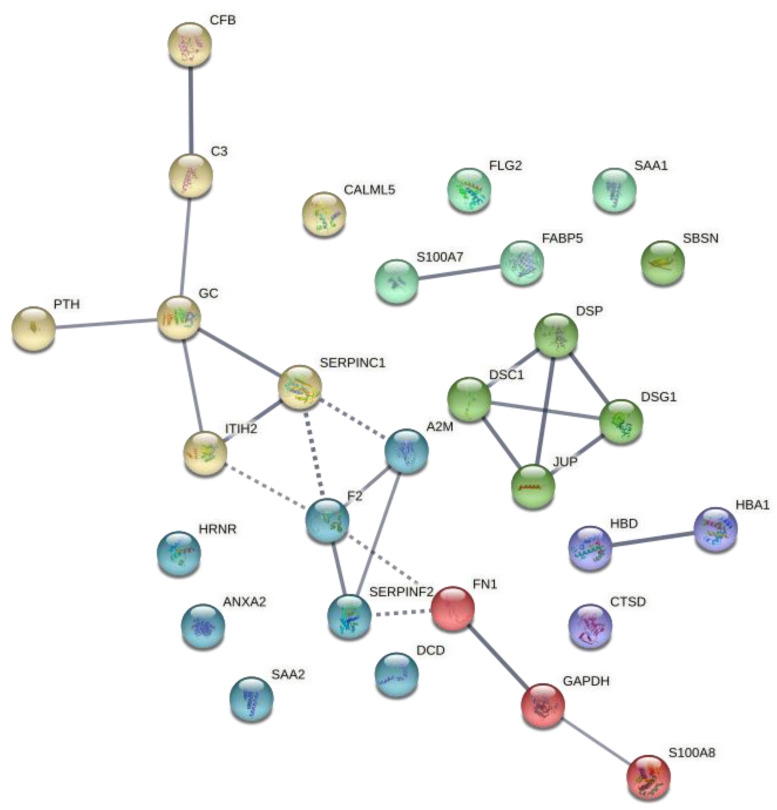
Protein–protein interaction network of the differentially expressed proteins after 6-month treatment with teriparatide. The edges show protein–protein interactions with predicted functional partners. Edges have different colors according to their clustering with k means with input number 6. All lines represent known protein–protein interactions with high confidence. Dot lines show high confidence interactions between different clusters.

Gene Ontology analyses with the list of genes displayed in Table 1 revealed enrichment in several immune-related pathways when using terms from biological processes (Table 2). Furthermore, after adding PTH as another relevant gene to evaluate all interactions, a string network was constructed with the list of proteins differentially expressed (Figure 2).

**Figure 3:**
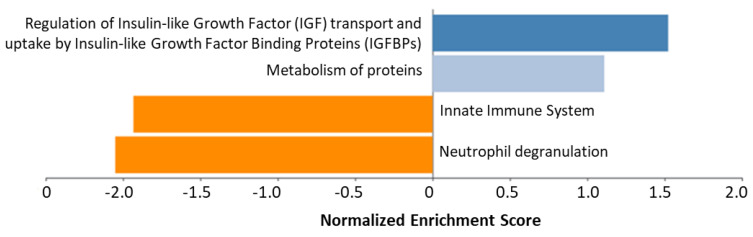
Gene-set enrichment analysis with the differentially expressed proteins after 6-month therapy with teriparatide. Orange bars to the left indicate downregulation of the pathway (FDR < 0.05), whereas blue bars to the right are represent upregulating effect on the pathway. Light blue is not significant (FDR > 0.05), whereas dark blue is significant (FDR < 0.05).

Gene-Set enrichment analysis was carried out by using the REACTOME tool, entering the 28 differentially expressed proteins. The top most significant pathways are shown in Figure 3. The “Regulation of Insulin-like Growth Factor (IGF) transport and uptake by Insulin-like Growth Factor Binding Proteins (IGFBPs)” pathway was significantly upregulated by a positive influence on the levels of the FN1, F2, C3, SERPINC1, and ITIH2 (Figure 3). On the contrary, the innate immune system and neutrophil degranulation pathways were downregulated. In this case, it should be noted that, although the protein levels are lower, the IGF regulation pathway is positive.

### 2.2. Denosumab

**Figure 4:**
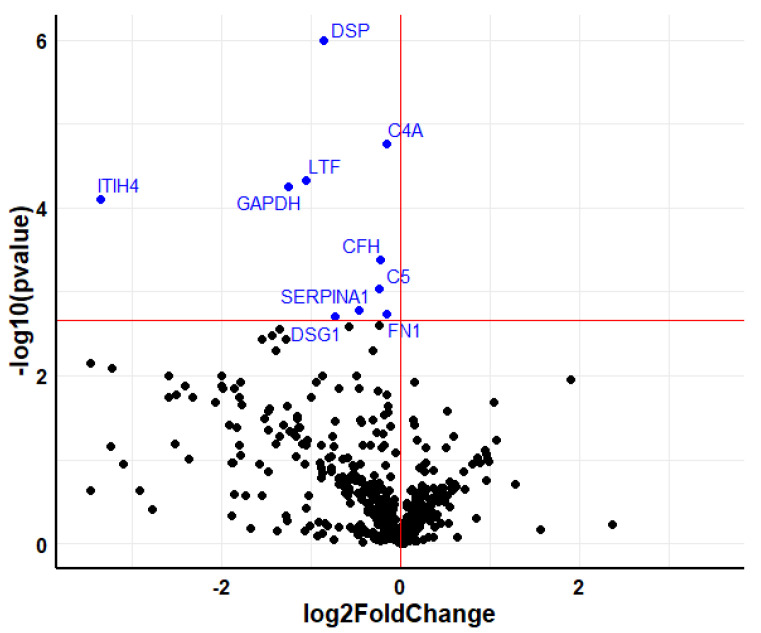
Volcano plot showing the differentially expressed proteins in serum after 6-month treatment with denosumab. The downregulated proteins are colored in blue. Horizontal red line marks the q-value threshold (0.05). Black circles are those proteins under the q-value threshold (not significant).

In the serum samples of the patient group treated with denosumab, 538 proteins were identified. After excluding keratins and apolipoproteins, 10 proteins showed significant differences in the comparison of pre- and posttreatment levels (Figure 4).

The genes encoding the proteins with significant differences pre- and post-therapy, ordered by q value, are shown in Table 3.

**Figure 5:**
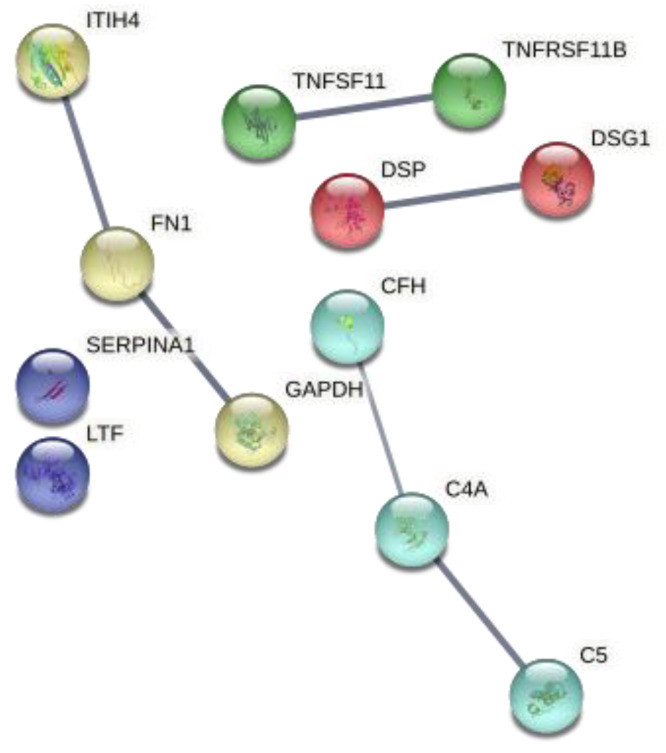
Protein–protein interaction network of the differentially expressed proteins after 6-month therapy with denosumab. The edges show protein–protein interactions with predicted functional partners. Edges have different colors according to their clustering with k means with input number 5. All lines represent known protein–protein interactions with high confidence.

Gene Ontology analyses with the list of genes shown in Table 3 also revealed enrichment in immune-related (Table 4). A string interaction network was constructed with this list of genes, after adding TNFSF11 and TNFRSF11B as other genes in the network (Figure 5). TNFSF11 and TNFRSF11B encode RANKL and osteoprotegerin (OPG), respectively. RANKL is the osteoclast-differentiating cytokine that is neutralized by denosumab, whereas OPG is a decoy of the receptor for RANKL and thereby inhibits osteoclastogenesis. Thus, OPG and RANKL are important components of the RANKL-RANK-OPG pathway that is targeted by denosumab.

**Figure 6:**
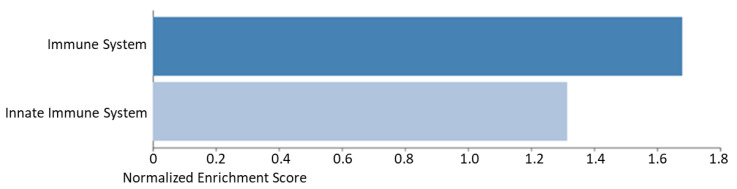
Gene-set enrichment analysis with the significant differentially expressed proteins after 6- month treatment with denosumab. Blue bars to the right are showing upregulating effects to the specific pathway. Light blue is not significant (FDR > 0.05), whereas dark blue is significant (FDR < 0.05).

In the Gene-Set enrichment analysis with REACTOME, the immune system emerged as a significant pathway. In contrast with the negative changes observed with teriparatide, this pathway appeared upregulated after denosumab therapy (Figure 6).

### 2.3. Analysis of Individual Proteins

**Figure 7:**
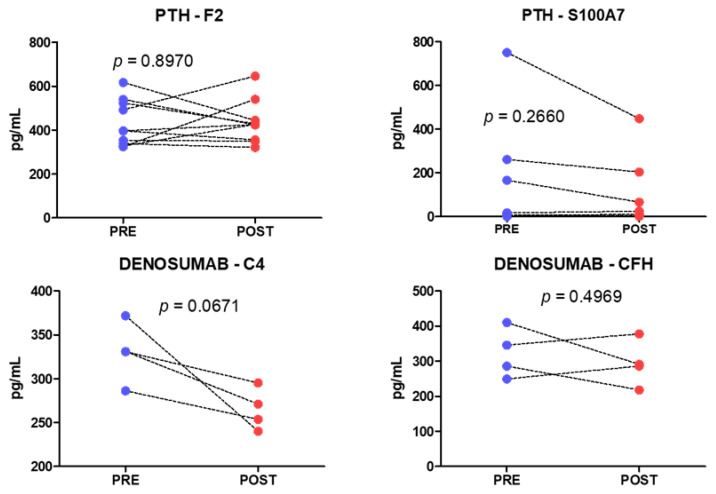
Serum levels of individual proteins in patients treated with teriparatide (**above**) or denosumab (**below**). *p*-values correspond to paired *t*-test analyses.

From each group of treatment, two markers were chosen to replicate F2 and S100A7 for the group treated with teriparatide (*n* = 10); and for the denosumab group (*n* = 4) C4 and CFH. A high interindividual variability was obtained in these analyses and no significant changes were observed in any of the markers (Figure 7).

## 3. Discussion

The results of this study suggest that drugs used to treat osteoporosis may impact the immune system, with changes that could be just epiphenomena without biological consequences or perhaps are involved in mediating drug effects.

A variety of studies in the last two decades have revealed the complex interactions between the immune system and the skeleton that had been encompassed under the term “osteoimmunology” [6]. Thus, cytokines and other products of immune cells influence bone cells [7,8], and the individual variation in immune responses has been associated with the risk of osteoporosis-related fractures [9]. In turn, osteoblasts and other skeletal cells play a critical role in maintaining “niches” suitable for the development of immune and hematopoietic cells [10,11]. Additionally, several tantalizing preclinical experiments in recent years suggest that gut microbiota may also impact the immune system and skeletal homeostasis, thus building a multilayer network of relationships between microbiota, immune cells, and skeletal cells [12,13,14].

The mechanisms explaining the anabolic effect of PTH (and the related molecules teriparatide and abaloparatide) are incompletely understood, but they likely involve multiple pathways. Preclinical data suggest that the osteoanabolic actions of PTH are due to directs effects on cells of the osteoblast lineage and to indirect effects mediated by stimulating IGF-I synthesis and suppressing the Wnt inhibitor sclerostin [15]. Not only bone cells, but also some cells of the immune system, such as several T cell populations and macrophages, express PTH receptors. Teriparatide induces an expansion of regulatory T cells (Tregs) in humans and mice and these cells seem to participate in the anabolic effect of PTH in murine models of osteoporosis [16]. On the other hand, in line with the role of the microbiota–immune axis, experiments showing that gut microbiota is involved in the anabolic response to PTH are particularly intriguing [17,18]. Thus, although the actual effect of endogenous PTH and exogenous teriparatide and other PTH-related molecules on immune function is still unclear, those experimental data and the results of the present study support a role of immune cells in the skeletal effects of bone anabolic drugs.

Denosumab is a RANKL-neutralizing antibody. RANKL binds its receptor RANK, expressed in osteoclast precursors, thus inducing their differentiation into mature bone-resorbing osteoclasts [19]. Hence, denosumab causes a marked inhibition in bone resorption, increases BMD, and reduces fracture risk [20,21]. Besides its role in osteoclast differentiation, RANKL is a costimulatory molecule involved in immune responses. However, unlike its critical role in osteoclast differentiation, RANKL does not seem to be essential for immune responses. In fact, infections and other immune-system-related adverse effects are very rare among denosumab-treated patients [22]. Nevertheless, this proteomic analysis also pointed out some immune-system-related proteins as impacted by denosumab therapy, a result that merits further investigation.

This study has several limitations. First, the sample size was rather small, particularly in the denosumab group. Additionally, although significant, the fold changes of differentially expressed proteins were small and their biological relevance has not been elucidated yet. We tried to measure serum levels of some proteins by immunoassay, but the results showed large interindividual dispersion and we were unable to confirm the differences in protein expression. Therefore, these results should be considered exploratory and require confirmation in larger series of patients and by using different protein-specific analyses. In this regard, techniques such as proximity extension analysis, may combine high throughput with high specificity. Additionally, specific analysis of immune mediators is needed to further delineate the pathways targeted by anabolic and antiresorptive agents. It would also be interesting to analyze serum protein changes at different time points, and explore the correlation, if any, between changes in these new markers and drug response, as evaluated by changes in DXA or in BTM levels.

## 4. Materials and Methods

Sample collection and preparation: Blood samples from 14 women with osteoporosis were obtained at baseline and 6 months after initiating therapy with either teriparatide (Forsteo^r^, Lilly, Indianapolis, IN, USA, 20 µg/day, *n* = 10) or denosumab (Prolia^r^ 60 mg, Amgen, Thousand Oaks, CA, USA, *n* = 4) (Table 5). Patients with secondary osteoporosis were excluded. After centrifugation for 10 min at 3000× *g*, serum aliquots were frozen until analysis. The study was approved by the institutional review board and all patients gave informed written consent.

Spin-columns High Select HSA/IG Depletion (Thermo, Waltham, MA, USA) were used to deplete albumin and IgG proteins, which are the major proteins in serum samples. Then, a digestion buffer (7 M Urea, 2 M Thiourea, 100 mM TEAB and 5% SDS) was used to denaturate the proteins and quantify by the PIERCE method at 660 nm. In addition, protein digestion with the S-Trap columns (Protifi, Farmingdale, NY, USA), following the manufacturer’s instructions. Digestions are quantified by fluorimetry (Qubit) to use a total of 1 µg per sample.

Liquid chromatography coupled to triple-TOF mass spectrometry: A measure of 1 µg aliquot of each digested sample was subjected to 1D-nano LC ESI-MSMS analysis using a nanoliquid chromatography system (Eksigent Technologies nanoLC Ultra 1D plus, SCIEX, Foster City, CA, USA) coupled to high-speed triple-TOF 5600 mass spectrometer (SCIEX, Foster City, CA, USA) with a Nanospray III source. The analytical column used was a silica-based reverse-phase Acquity UPLC M-Class Peptide BEH C18 Column, 75 µm × 150 mm, 1.7 µm particle size and 130 Å pore size (Waters, Milford, MA, USA). The trap column was a C18 Acclaim PepMapTM 100 (Thermo Scientific), 100 µm × 2 cm, 5 µm particle diameter, 100 Å pore size, switched online with the analytical column. The loading pump delivered a solution of 0.1% formic acid in water at 2 µL/min. The nano-pump provided a flow rate of 250 nL/min and was operated under gradient elution conditions. Peptides were separated using a 250-minute gradient ranging from 2 to 90% mobile phase B (mobile phase A: 2% acetonitrile, 0.1% formic acid; mobile phase B: 100% acetonitrile, 0.1% formic acid). Injection volume was 5 µL. Data acquisition was performed with a triple-TOF 5600 System (SCIEX, Foster City, CA, USA). Data was acquired using an ionspray voltage floating (ISVF) 2300 V, curtain gas (CUR) 35, interface heater temperature (IHT) 150, ion source gas 1 (GS1) 25, declustering potential (DP) 100 V. All data was acquired using information-dependent acquisition (IDA) mode with Analyst TF 1.7 software (SCIEX, USA). For IDA parameters, 0.25 s MS survey scan in the mass range of 350–1250 Da were followed by 35 MS/MS scans of 100 ms in the mass range of 100–1800 (total cycle time: 4 s). Switching criteria were set to ions greater than mass to charge ratio (*m*/*z*) 350 and smaller than *m*/*z* 1250 with charge state of 2–5 and an abundance threshold of more than 90 counts (cps). Former target ions were excluded for 15 s. IDA rolling collision energy (CE) parameters script was used for automatically controlling the CE.

Data analysis and quantification: The mass spectrometry data obtained were processed using PeakView^®^ 2.2 Software (SCIEX, Foster City, CA, USA) and exported as mgf files. Proteomics data analysis were performed by using 4 search engines (Mascot Server v.2.5.1, OMSSA, X!Tandem and Myrimatch) and a target/decoy database built from sequences in the Homo sapiens proteome at Uniprot Knowledgebase.

All search engines were configured to match potential peptide candidates to recalibrated spectra with mass error tolerance of 10 ppm and fragment ion tolerance of 0.02 Da, allowing for up to 2 missed tryptic cleavage sites and a maximum isotope error (13C) of 1, considering fixed MMTS modification of cysteine and variable oxidation of methionine, pyroglutamic acid from glutamine, or glutamic acid at the peptide N-terminus. Score distribution models were used to compute peptide-spectrum-matched *p*-values [23], and spectra recovered by an FDR ≤ 0.01 (peptide-level) filter were selected for quantitative analysis. Differential regulation was measured using linear models [24], and statistical significance was measured using q-values (FDR). All analyses were conducted using software from Proteobotics (Madrid, Spain).

Gene ontology analysis was performed with Webgestalt over-representation analyses by using non-redundant biological process and non-redundant molecular function. Interactions between differentially expressed proteins and pathway enrichment analyses were carried out by using the STRING database (Search Tool for the Retrieval of Interacting Genes/Proteins) and the Webgestalt and Reactome web tools, respectively [25,26,27]. For the STRING-based analyses, the number of k means cluster was set to 6 and the interaction score to high confidence.

Analysis of individual proteins: From the teriparatide group S100A7 and F2 proteins were measured in the previously obtained serum samples by ELISA, accordingly to manufacturers’ instructions (Thermo Fisher Scientific, Waltham, MA, USA), whereas in the denosumab group, two complement factors (C4 and CFH) were measured with the Human Complement Panel 2 Bead-Based Multiplex Assay kit (MERCK, Darmstadt, Germany).

## 5. Conclusions

This analysis of serum peptides suggests that both bone-forming and antiresorptive therapies impact the immune system. While the pathogenic relevance of these findings remains to be elucidated, they are worthwhile pursuing. Further investigations in this line may be important not only for a better understanding of the mechanisms involved in the effects of drugs on bone, but also as a way to potentially find novel therapeutic approaches, particularly important regarding bone anabolic therapies.

## Figures and Tables

**Table 1:** Differentially expressed proteins in serum after 6-month treatment with teriparatide, ordered by q-value.

Gene Symbol	Gene Full Name	Log_2_ Fold Change	q-Value
FN1	Fibronectin	−0.241	1.3 × 10^−5^
F2	Prothrombin F2	−0.173	3.9 × 10^−5^
SERPINF2	Alpha-2-antiplasmin	−0.219	1.0 × 10^−3^
C3	Complement C3	−0.049	1.0 × 10^−3^
ANXA2	Annexin A2	−1.024	4.0 × 10^−3^
HRNR	Hornerin	−0.973	4.0 × 10^−3^
JUP	Junction plakoglobin	-0.81	4.0 × 10^−3^
DSP	Desmoplakin	−0.756	4.0 × 10^−3^
S100A7	Protein S100-A7	−1.262	5.0 × 10^−3^
GC	Vitamin D-binding protein	−0.099	6.0 × 10^−3^
A2M	Alpha-2-macroglobulin	0.093	6.0 × 10^−3^
GAPDH	Glyceraldehyde-3-phosphate dehydrogenase	−1.353	1.0 × 10^−2^
FABP5	Fatty acid-binding protein 5	−2.106	1.2 × 10^−2^
CALML5	Calmodulin-like protein 5	−1.447	1.2 × 10^−2^
FLG2	Filaggrin-2	−0.928	1.3 × 10^−2^
DSG1	Desmoglein-1	−1.067	1.6 × 10^−2^
DSC1	Desmocollin-1	−0.919	2.0 × 10^−2^
CTSD	Cathepsin D	−0.901	2.1 × 10^−2^
SBSN	Suprabasin	−0.62	2.2 × 10^−2^
S100A8	Protein S100-A8	−0.852	2.6 × 10^−2^
SAA1	Serum amyloid A-1 protein	0.764	3.1 × 10^−2^
SERPINC1	Antithrombin-III	−0.109	3.2 × 10^−2^
SAA2	Serum amyloid A-2 protein	2.684	3.3 × 10^−2^
HBD	Hemoglobin subunit delta	−0.517	3.3 × 10^−2^
ITIH2	Inter-alpha-trypsin inhibitor heavy chain H2	−0.103	3.5 × 10^−2^
CFB	Complement factor B	0.09	4.0 × 10^−2^
DCD	Dermcidin	−0.63	4.3 × 10^−2^
HBA1	Hemoglobin subunit alpha	−0.472	4.3 × 10^−2^

**Table 2:** Gene Ontology analyses of the differentially expressed proteins after 6-month treatment with teriparatide.

**Biological Process**			
**Gene Set**	**Description**	**FDR**	**Gene ID**
GO:0002446	Neutrophil mediated immunity	1.06 × 10^−11^	F2; C3; ANXA2; HRNR; JUP; DSP; S100A7; FABP5; CALML5; FLG2; DSG1; DSC1; CTSD; S100A8
GO:0002526	Acute inflammatory response	2.16 × 10^−11^	FN1; F2; SERPINF2; C3; A2M; S100A8; SAA1; SERPINC1; SAA2; CFB
GO:0006959	Humoral immune response	1.17 × 10^−6^	F2; C3; S100A7; A2M; GAPDH; S100A8; CFB; DCD
GO:0052547	Regulation of peptidase activity	6.40 × 10^−5^	FN1; SERPINF2; C3; A2M; GAPDH; S100A8; SERPINC1; ITIH2
**Molecular Function**			
**Gene Set**	**Description**	**FDR**	**Gene ID**
GO:0061134	Peptidase regulator activity	4.81 × 10^−6^	FN1; SERPINF2; C3; A2M; GAPDH; SERPINC1; ITIH2
GO:0098631	Cell adhesion mediator activity	6.74 × 10^−3^	ANXA2; JUP; DSP
GO:0017171	Serine hydrolase activity	1.41 × 10^−2^	F2; C3; CTSD; CFB
GO:0005539	Glycosaminoglycan binding	1.43 × 10^−2^	FN1; F2; SAA1; SERPINC1

**Table 3:** Differentially expressed proteins in serum after 6-month treatment with denosumab, ordered by q-value.

Gene Symbol	Gene Full Name	Log_2_ Fold Change	q-Value
DSP	Desmoplakin	−0.856	3.2 × 10^−5^
C4A	Complement C4-A	−0.157	1.0 × 10^−3^
LTF	Lactotransferrin	−1.057	2.0 × 10^−3^
ITIH4	Inter-alpha-trypsin inhibitor heavy chain H4	−3.358	3.0 × 10^−3^
GAPDH	Glyceraldehyde-3-phosphate dehydrogenase	−1.253	3.0 × 10^−3^
CFH	Complement factor H	−0.224	1.3 × 10^−2^
C5	Complement C5	−0.238	2.4 × 10^−2^
SERPINA1	Alpha-1-antitrypsin	−0.47	4.2 × 10^−2^
FN1	Fibronectin	−0.159	4.3 × 10^−2^
DSG1	Desmoglein-1	−0.734	4.5 × 10^−2^
DSP	Desmoplakin	−0.856	3.2 × 10^−5^

**Table 4:** Gene Ontology analyses of the differentially expressed proteins after 6-month treatment with denosumab.

**Biological Process**			
**Gene Set**	**Description**	**FDR**	**Gene ID**
GO:0002526	Acute inflammatory response	1.98 × 10^−7^	C4A; ITIH4; CFH; C5; SERPINA1; FN1
GO:0052547	Regulation of peptidase activity	6.44 × 10^−7^	C4A; LTF; ITIH4; GAPDH; C5; SERPINA1; FN1
GO:0002446	Neutrophil mediated immunity	1.95 × 10^−2^	DSP; LTF; SERPINA1; DSG1
**Molecular Function**			
**Gene Set**	**Description**	**FDR**	**Gene ID**
GO:0061134	Peptidase regulator activity	3.98 × 10^−9^	C4A; LTF; ITIH4; GAPDH; C5; SERPINA1; FN1
GO:0043394	Proteoglycan binding	1.96 × 10^−2^	CFH; FN1

**Table 5:** Patients characteristics.

Lab Code	Treatment	Sex	Age	T-Score Lumbar Spine	T-Score Femoral Neck	Fractures	Treatment in Previous 12 Months	BMI
P1	Teriparatide	Female	89	−4.9	−3.1	Vertebral	None	27.5
P2	Teriparatide	Female	63	−0.8	−0.7	Vertebral	None	30.5
P3	Teriparatide	Female	65	−2.8	−1.6	Vertebral	None	28.8
P4	Teriparatide	Female	79	−0.9	−2.2	Vertebral, peripheral	Denosumab	27.4
P5	Teriparatide	Female	81	-	-	Vertebral	None	33.3
P6	Teriparatide	Female	84	−2.6	−2.3	Vertebral, peripheral	None	28.1
P7	Teriparatide	Female	91	−1.4	−3.7	Vertebral	None	24.5
P8	Teriparatide	Female	77	−1.4	−2.1	Peripheral	None	28.8
P9	Teriparatide	Female	79	−4.2	−2.7	Vertebral	None	20.4
P10	Teriparatide	Female	78	−3.3	−2.7	Vertebral	None	21.5
D1	Denosumab	Female	80	−1.4	−0.8	Vertebral	None	21.9
D2	Denosumab	Female	80	-	-	Vertebral	None	28.2
D3	Denosumab	Female	72	−2.7	−2.5	Vertebral	None	22.7
D4	Denosumab	Female	79	−2.9	−3.6	Vertebral	None	23.9

## Data Availability

Raw data are available from authors upon reasonable request due to the nature of data.
